# Impact of hypertension history and blood pressure parameters on cognitive impairment in patients with atrial fibrillation: a systematic review and meta-analysis

**DOI:** 10.3389/fcvm.2026.1820778

**Published:** 2026-06-02

**Authors:** Xingxing Mi, Ting Wang, Weidong Ye, Yamei Yuan

**Affiliations:** 1 School of Nursing, Anhui University of Chinese Medicine, Hefei, Anhui, China; 2 Key Laboratory of Geriatric Nursing and Health, Anhui University of Chinese Medicine, Hefei, Anhui, China; 3 Nursing Department, Second Affiliated Hospital of Anhui University of Chinese Medicine, Hefei, Anhui, China; 4 School of Traditional Chinese Medicine, Anhui University of Chinese Medicine, Hefei, Anhui, China

**Keywords:** atrial fibrillation, cognitive impairment, hypertension, blood pressure, influencing factors, systematic review, meta-analysis

## Abstract

**Background:**

Cognitive impairment manifests as a gradual decline in the ability to perform daily activities. This study evaluates the impact of history of hypertension and blood pressure parameters on cognitive impairment in patients with atrial fibrillation (AF).

**Methods:**

We systematically searched eight databases (CNKI, Wanfang, VIP, CBM, PubMed, Embase, Cochrane Library, Web of Science) from inception to September 7, 2025, for observational studies examining associations between hypertension history, blood pressure parameters, and cognitive impairment in AF patients. Study quality was assessed using NOS (cohort/case-control) and AHRQ (cross-sectional) tools. Meta-analyses were performed separately for odds ratios (OR) and hazard ratios (HR) using RevMan 5.4. Heterogeneity was evaluated with I^2^ statistic, with sensitivity and subgroup analyses.

**Results:**

A total of 20 studies involving 998,622 AF patients were included. We identified six potential risk factors for cognitive impairment in patients with atrial fibrillation from these 20 studies, three of which (history of hypertension, systolic blood pressure, diastolic blood pressure) were reported in sufficient studies to be included in a meta-analysis. Subgroup meta - analysis of OR showed that history of hypertension is associated with an increased risk of cognitive impairment in patients with atrial fibrillation (OR: 2.22, 95% CI: 2.15–2.30, *P* < 0.00001; I^2^= 4%).Further subgroup analyses indicated association between history of hypertension and the risk of mild cognitive impairment (OR = 2.06, 95% CI: 1.10–3.85, *P* = 0.02; I^2^= 59%), and between history of hypertension and overall cognitive impairment risk (OR = 2.22, 95% CI: 2.15–2.30, *P* < 0.00001; I^2^= 0%). Additionally, history of hypertension may increase the risk of dementia (OR = 4.88, 95% CI: 1.29–18.45, *P* = 0.02).The HR subgroup meta - analysis showed that high - normal blood pressure levels (130 - 139/85 - 89 mmHg) were significantly associated with cognitive impairment in AF patients (HR: 1.07, 95% CI: 1.01–1.13, *P* = 0.02; I^2^= 0%).

**Conclusion:**

History of hypertension and high-normal blood pressure levels are significantly associated with cognitive impairment in patients with AF. Identifying and intervening in these modifiable risk factors is crucial for prevention and treatment.

**Systematic Review Registration:**

https://www.crd.york.ac.uk/PROSPERO/view/CRD420251186104, PROSPERO CRD420251186104.

## 1 Introduction

Atrial fibrillation (AF), as one of the most common clinical arrhythmias, has witnessed a continuous increase in global prevalence, emerging as a significant public health burden worldwide. It mainly presents with cardiovascular complications, a significantly elevated risk of brain injury, and is closely associated with the incidence of cognitive impairment (1, 2). Cognitive impairment is a neuropsychiatric syndrome characterized by a progressive decline in the ability to carry out daily activities, which may ultimately progress to dementia or Alzheimer's disease in the later stages (3). The relationship between AF and cognitive impairment involves multiple complex mechanisms, including hemodynamic disturbances and asymptomatic cerebral ischemia. Hypertension, as a common and modifiable risk factor for both conditions, plays a crucial role in their etiology and pathophysiological processes (4). In the general population, the correlation between blood pressure levels and cognitive outcomes has been well-established (5). However, in the specific population of AF patients, the impact of a history of hypertension and specific blood pressure parameters on cognitive function has not been systematically clarified, owing to confounding factors such as hemodynamic disturbances and the use of anticoagulants. Additionally, previous studies have demonstrated significant heterogeneity in the effect indicators used: cross-sectional or case-control studies frequently employ odds ratios (OR), whereas cohort studies utilize hazard ratios (HR), which differ notably in clinical interpretation and statistical characteristics. Based on this, the present study aims to adopt a stratified analysis approach to systematically evaluate the association between a history of hypertension and blood pressure parameters, as well as cognitive impairment in AF patients, providing high-quality evidence to guide the development of precise cognitive function protection strategies in clinical practice.

## 2 Methods

This study was registered in the international prospective register of systematic reviews (PROSPERO registration number(CRD420251186104) and was carried out in accordance with the PRISMA guidelines for Systematic Reviews and Meta-Analyses.

### 2.1 Search strategy

Researchers systematically searched eight databases (CNKI, VIP, China Biomedical Literature Database, Wanfang Data Knowledge Service Platform, PubMed, Embase, Web of Science, Cochrane Library) for studies on the effects of a history of hypertension and blood pressure parameters on cognitive impairment in patients with atrial fibrillation. They also tracked the references of the included studies. The search period ranged from the establishment of the databases to September 7, 2025. Search terms in Chinese databases included both subject terms and free-text terms, such as atrial fibrillation, AF, persistent atrial fibrillation, paroxysmal atrial fibrillation, auricular fibrillation, cognitive dysfunction, mild cognitive impairment, cognitive decline, and cognitive damage. For English databases, the search combined MeSH terms like ‘Atrial Fibrillation’ and its derivatives ‘AF,’ ‘Atrial Flutter,’ with cognitive dysfunction-related terms such as ‘Cognitive Impairment,’ ‘Cognitive Decline,’ ‘MCI,’ and ‘Dementia.’ Taking PubMed as an example, the detailed search strategy is shown in Table 1. The complete search strategies for all databases are presented in Supplementary Table S1.

**Table 1:** Pubmed search strategy.

PubMed search strategy	
Search step	Search Terms
#1	MeSH tems:"Cognitive Dysfunction" OR "Dementia"
#2	Title/Abstract:"MCI” OR "mild cognitive impairment” OR "Cognitive Dysfunction” OR "cognitive impairment” OR "cognitive decline"OR "neurocognitive disorders” OR "Dementia” OR "alzheime disease” OR "Cognition” OR "MMSE” OR "Dementias, Vascular” OR "Arteriosclerotic Dementia” OR "Chronic Progressive Subcortical Encephalopathy” OR "Diffuse Lewy Body Disease” OR "Frontotemporal Lobe Dementias " OR "Mental Disorders, Organic” OR "Organic Mental Disorders, Psychotic” OR "MCI, Dementia"
#3	#1 OR#2
#4	MesH tems:"Atrial Fibrillation"
#5	Title/Abstract:"fibrillation atrial"OR"fibrillations atrial"OR "auricular fibrillation” OR"atrial flutter"OR"AFib"OR "AF"OR "atrial fibril*"OR"auricular fibril*"
#6	#4 OR #5
#7	#3 AND #6

### 2.2 Inclusion and exclusion criteria

#### 2.2.1 Inclusion criteria

(1) Study Population: Patients with a confirmed diagnosis of atrial fibrillation, including paroxysmal atrial fibrillation and familial atrial fibrillation. (2) Study Types: Cohort studies, cross-sectional studies, and case-control studies; different projects by the same researcher are also included. (3) Exposure Factors: History of hypertension or any blood pressure parameters. (4) Study Data: Studies providing adjusted OR values, HR values, and their 95% confidence intervals. (5) Outcome Measures: Cognitive impairment, dementia, or mild cognitive impairment. (6) Study Language: Only studies in Chinese and English are included.

#### 2.2.2 Exclusion criteria

(1) Full text not accessible. (2) Duplicate literature (only one version selected). (3) Animal studies, conference papers, reviews, case reports, dissertations. (4) Incomplete data.

### 2.3 Literature screening and data extraction

The retrieved literature was imported into EndNote 21 for duplicate removal. Two researchers independently screened the literature and extracted data according to predefined inclusion and exclusion criteria. All results were cross-checked, and any disagreements were resolved through discussion or consultation with a third researcher. The initial screening was conducted by reviewing titles and abstracts, followed by a full-text screening for potentially eligible studies. For studies meeting the criteria, information such as the first author, year of publication, country, study design, sample size, and specific exposure variables was extracted.

### 2.4 Literature quality assessment

The quality of cohort and case-control studies was evaluated using the Newcastle-Ottawa Scale (NOS), which encompasses three domains: selection of study groups, comparability between groups, and assessment of exposure or outcome. The NOS assigns a total score ranging from 0 to 9, with scores of 0–4 classified as low quality, 5–6 as medium quality, and 7–9 as high quality (6). Cross-sectional studies were assessed using the risk of bias tool recommended by the Agency for Healthcare Research and Quality (AHRQ), which includes 11 items (7). Each item is scored as 1 point for "yes” and 0 points for "no” or "unclear”; a total score≤3 indicates low quality, 4-7indicates medium quality, and ≥8 indicates high quality. Two researchers independently conducted the quality assessments, and any discrepancies were resolved through discussion or, when necessary, by consultation with a third researcher.

### 2.5 Statistical analysis

The study initially identified six potential risk factors, but due to limited reported data, only three factors (history of hypertension, systolic blood pressure, diastolic blood pressure) were reported in a sufficient number of studies and could be included in the meta-analysis. Using RevMan 5.4 software, the odds ratios (ORs), hazard ratios (HRs), and 95% credible intervals (CIs) of cognitive dysfunction in patients with atrial fibrillation for history of hypertension and blood pressure parameters (systolic and diastolic blood pressure) were calculated. In view of the clinical heterogeneity between ORs and HRs, they were combined separately. First, the combination of OR values: the effect of a history of hypertension on cognitive impairment; the effect of systolic/diastolic blood pressure on cognitive impairment. Second, the combination of HR values: the effect of a history of hypertension on the long-term risk of cognitive impairment; the effect of systolic/diastolic blood pressure on the long-term risk of cognitive impairment. I^2^ was used to assess heterogeneity in the combined data. If I^2^ < 50% and *P* > 0.05, it indicates that the heterogeneity among studies is acceptable, and a fixed-effects model is used for analysis; if I^2^ ≥ 50% or *P* < 0.05, it indicates significant heterogeneity among studies, and a random-effects model is used for analysis. Sensitivity analysis was used to explore the impact of each included study on the combined effect value, and exploratory subgroup analyses were also conducted. For meta-analyses including ten or more studies, publication bias was assessed by evaluating the symmetry of funnel plot distribution.

## 3 Results

### 3.1 Study selection

**Figure 1:**
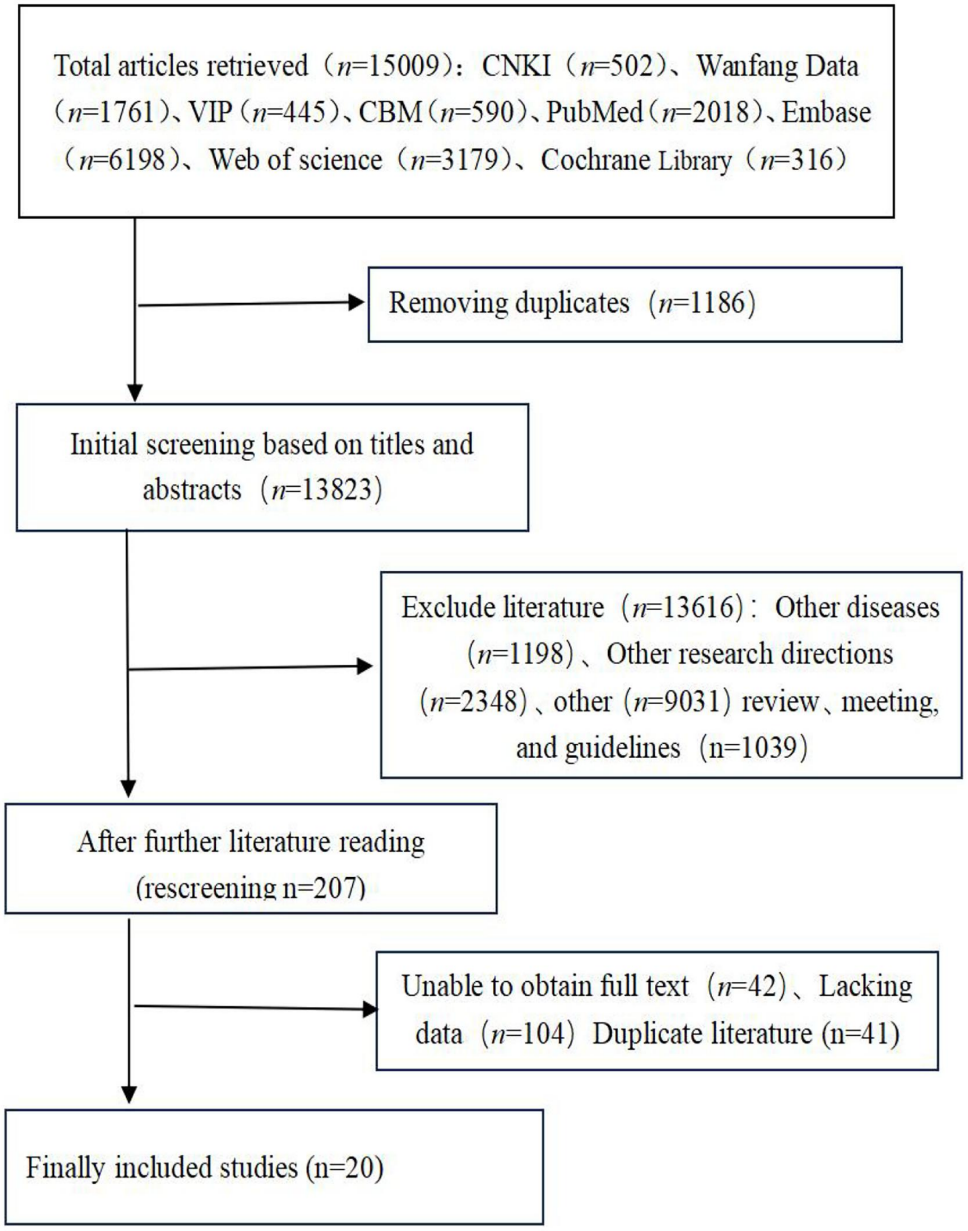
Literature screening flowchart.

A total of 15,009 relevant articles were found through database searches. Using EndNote 21, 1,186 duplicate articles were removed, and 13,823 irrelevant studies were excluded based on their titles and abstracts. Finally, 20 articles were selected according to the inclusion and exclusion criteria. The literature screening process is shown in Figure 1.

### 3.2 Basic characteristics and quality evaluation of the included literature

A total of 20 studies were included, involving 998,622 patients with atrial fibrillation. The types of studies comprised 3 case-control studies, 9 cohort studies, and 8 cross-sectional studies. In terms of geographic distribution, the studies were mainly conducted in China (12 studies; *n* = 5,788), followed by Switzerland (2 studies; *n* = 3,628), the United States (1 study; *n* = 621,773), the United Kingdom (1 study; *n* = 183,610), South Korea (1 study; *n* = 171,228), Sweden (1 study; *n* = 12,283), India (1 study; *n* = 108), and France (1 study; *n* = 204). All included cross-sectional studies had an AHRQ score of ≥ 4, and all included cohort and case-control studies had an NOS score of ≥ 6, indicating high study quality. The basic information of the included studies is summarized in Table 2

**Table 2:** Basic information and quality assessment of included literature.

Author(year)	Region	Study type	Sample size	Hypertension n (%)	Assessment tool	outcome	Exposure definition	study descriptors	Key adjustment variables	Influence factor	Quality score
Xu (8) 2017	China	Cross-sectional study	303	193 (63.7)	MMSE	MCI	/	Hospitalized AF patients	a	1,2	4^a^
Xie (19) 2023	China	Cross-sectional study	143	101 (70.62)	MMSE	CD	/	Hospitalized AF patients	b	2	4^a^
He (9) 2022	China	Case-control study	198	69 (34.85)	/	Dementia	/	Hospitalized AF patients	c	1	6^b^
Kong (10) 2024	China	Cross-sectional study	2294	1427 (62.2)	MoCA	MCI	Blood≥140/90 mmHg	Hospitalized AF patients	d	1	8^a^
Liu (11) 2021	China	Cohort study	113	33 (29.2)	Guide	Dementia	/	Hospitalized AF patients	e	1	8^b^
Wang (12) 2021	China	Cross-sectional study	106	64 (60.38)	Guide	MCI	/	Hospitalized AF patients	f	1	6^a^
Yang (13) 2021	China	Case-control study	104	89 (85.58)	MoCA	MCI	/	Hospitalized AF patients	g	1	8^b^
Bian (39) 2023	China	Cross-sectional study	121	42 (34.71)	CDR	CD	/	Hospitalized AF patients	h	1	6^a^
Feng (20) 2024	China	Cross-sectional study	120	56 (46.67)	MoCA	CI	/	Hospitalized AF patients	i	2,3	6^a^
Xiong (22) 2019	China	Cross-sectional study	130	103 (79.23)	MMSE	CD	/	Hospitalized AF patients	j	3	6^a^
Lefebvre (15) 2025	France	Case-control study	204	152 (74.51)	IQCODE	dementia	antihypertensive drugs	Hospitalized AF patients	k	1	8^b^
Liang (16) 2025	China	Cohort study	2016	1118 (55.46)	Clinical evaluation	dementia	/	Hospitalized AF patients	l	1,2,3	8^b^
Batta (21) 2022	India	Cohort study	108	/	/	Dementia	/	care centre AF patients	m	2	7^b^
Zhao (17) 2024	China	Cross-sectional study	140	102 (72.86)	MMSE	CD	Blood≥140/90 mmHg	Hospitalized AF patients	n	1	4^a^
Wändell (18) 2018	Sweden	Cohort study	12283	5449 (44.36)	/	Dementia	/	75 primary health care centers	o	1	8^b^
Kim (26) 2020	Korea	Cohort study	171228	120172 (70.2)	International Classification of Diseases Code, Dementia Medications	Dementia	Blood≥140/90 mmHg	NHIS database	p	4,5,6	8^b^
Carmine (27) 2024	Switzerland	Cohort study	1213	821 (67.7)	MoCA	CD	self-reported/medical reports	Multicenter Swiss-AF study data	q	4,5,6	7^b^
Brooks (23) 2024	United Kingdom	Cohort study	183610	121,367 (66.1)	/	dementia	/	HDRUK	r	1	6^b^
Alam (24) 2022	United States	Cohort study	621773	436366 (70.18)	/	Dementia	/	IBM Watson	s	1	7^b^
Wueest (25) 2023	Switzerland	Cohort study	2415	1691 (70.02)	MoCA	CD	/	outpatient AF patients from 14 centers	t	1	8^b^

a = Agency for Healthcare Research and Quality, AHRQ; ^b^ = Newcastle Ottawa Scale,NOS;/=Not specified;.

Influence factor:

1 = History of hypertension; 2 = Systolic blood pressure; 3 = Diastolic blood pressure; 4High-normal blood pressure (130-139/85-89 mmHg); 5 = Systolic blood pressure (SBP) increases by 10 mmHg; 6 = Diastolic blood pressure (DBP) increases by 10 mmHg (starting from 80 mmHg).

Key adjustment variables (codes a–t):

a = Age, sex, education, CHA₂DS₂-VASc, AF type, heart failure, diabetes, stroke, left atrial diameter, fasting glucose; b = Age, coronary heart disease, red blood cell distribution width, rate control therapy; c = Diabetes, obesity, smoking, dyslipidemia, angina, anticoagulant drugs; d = Age, gender, education level, SDS, SAS, number of common cardiovascular diseases, having ≥3 common cardiovascular diseases; e = Age, heart failure, persistent permanent AF, novel oral anticoagulants; f = Persistent/permanent AF, type 2 diabetes, HGB, CHA2DS2-VASc score, gender; g = Atrial fibrillation, hyperlipidemia, age, educational level; h = Warfarin anticoagulation, diabetes, LVEF (left ventricular ejection fraction); i = Age、Occupational Situation、Education Level、Smoking History、Drinking History、Heart Failure、TC、TG、CRP、FT4、FT3、D-D; j = Age、Sex、Education、Anti-coagulation、Smoking history、NT-proB-type natriuretic peptide; k = Age、Education level ≥8 years、Paroxysmal NVAF、Previous ischemic stroke or TIA、Diabetes mellitus、Hyperlipidemia、Multiple infarcts、Leukoaraiosis; l = Female, Age, BMI, Heart rate, Type of AF, Comorbidities, LVH by ECG or echocardiogram, Previous stroke or TIA, Smoking,Left ventricular systolic dysfunction, Diabetes mellitus, Prior major bleeding; m = Age, female sex, presence of diabetes, a lower serum albumin; *n* = Age, education level, diabetes, and hyperuricemia; o = myocardial infarction, congestive heart failure, cerebrovascular diseases, obesity, diabetes, COPD, depression, and anxiety; *p* = Age, Male, Income, Body mass index, kg/m2, Heavy alcohol consumption, Current smoking, Physical activity ≥3 times/wk, Heart failure, Diabetes mellitus, Dyslipidemia, Prior ischemic stroke, Prior transient ischemic attack, Prior intracranial hemorrhage, Prior myocardial infarction, Peripheral artery disease, Chronic kidney disease, Chronic liver disease, Malignant neoplasm, Venous thromboembolism, CHA2DS2-VASc score, Hospital frailty risk score, Charlson comorbidity index, Medication use at baseline; q = geriatric depression score, body mass index, smoking status, previous stroke or transient ischemic attack, history of diabetes, history of heart failure, history of coronary heart disease, atrial fibrillation type, oral anticoagulation, antithrombotic treatment, and antihypertensive treatment; r = Sex, Ethnicity, Previous myocardial infarction, Previous heart failure, Abdominal aortic aneurysm, History of peripheral arterial disease, Previous intracerebral haemorrhage, History of diabetes, BMI, Alcohol, Smoker, Anti-coagulants, Non-steroidal anti-inflammatory drugs, Anti-platelets, Statins, Diuretics;s = Stroke, Heart Failure, Diabetes mellitus, Cerebral Bleeding，Hyperlipidemia, Coronary Heart Disease, Peripheral Artery Disease, Chronic Kidney Disease, VTE, Frailty, Alcohol Abuse, Anemia, Arthritis, Asthma, Cancer, Coagulopathy; sex, history of stroke, smoking status, or for patients with and without diabetes, or depression; t = sex, history of stroke, smoking status, or for patients with and without diabetes, or depression.

CD, cognitive disorder; CI, Cognitive Impairment; MMSE, Simplified Intelligent Status Checklist; MoCA, Montreal Cognitive Assessment; Guide, ‘2018 China Dementia and Cognitive Impairment Diagnosis and Treatment Guidelines' CDR, Cognitive disorder rating; IQCODE, (the Informant Questionnaire on Cognitive Decline in the Elderly); Clinical evaluation, Doctors use neuropsychological examinations, past medical history, imaging tests, and laboratory tests; HDRUK, Phenotype library validated by the UK Health Data Research Centre; IBM Watson, IBM Watson Ann Arbor, MIdatabase.

### 3.3 Meta-analysis results

We extracted six potential risk factors from 20 studies, including: history of hypertension, systolic blood pressure, diastolic blood pressure, high-normal blood pressure (130-139/85 - 89 mmHg), increase of 10 mmHg in systolic blood pressure, and increase of 10 mmHg in diastolic blood pressure. Among these, three factors (history of hypertension, systolic blood pressure, and diastolic blood pressure) reported odds ratios (OR) or hazard ratios (HR) in at least two studies, and were therefore included in the formal meta-analysis.

#### 3.3.1 History of hypertension: combined OR, sensitivity analysis, and publication bias assessment

**Figure 2:**
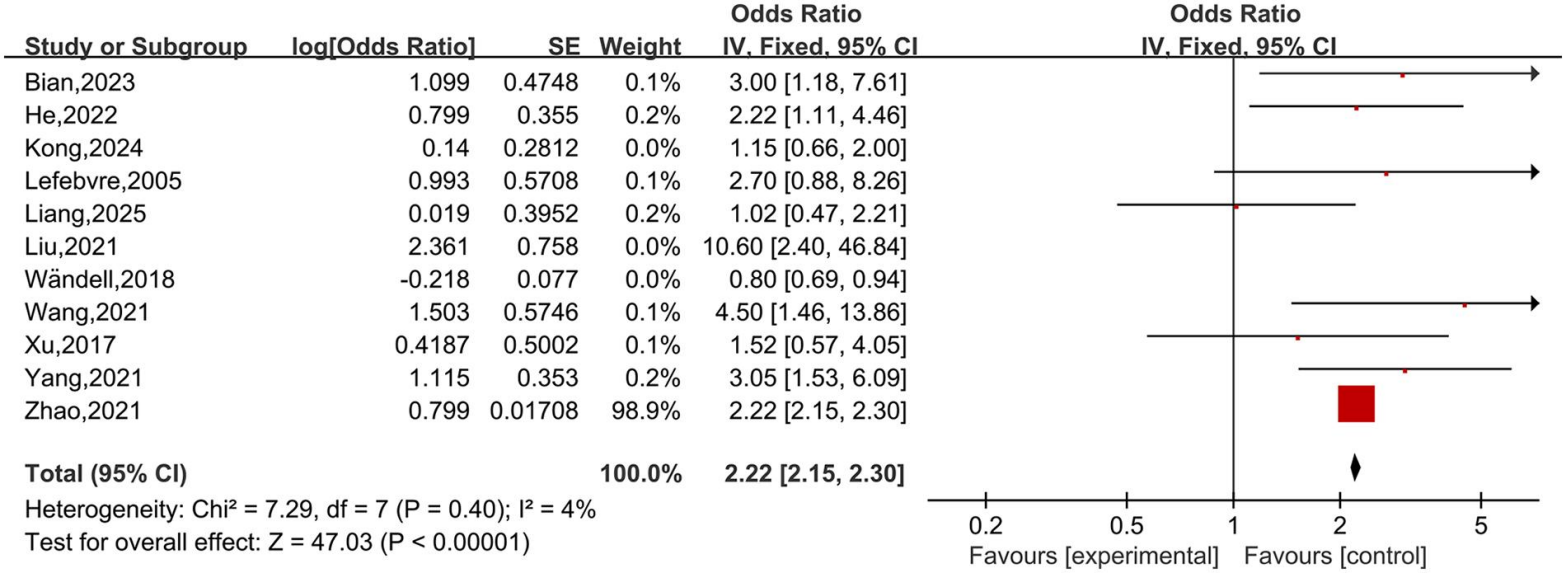
Hypertension history combined OR values.

**Figure 3:**
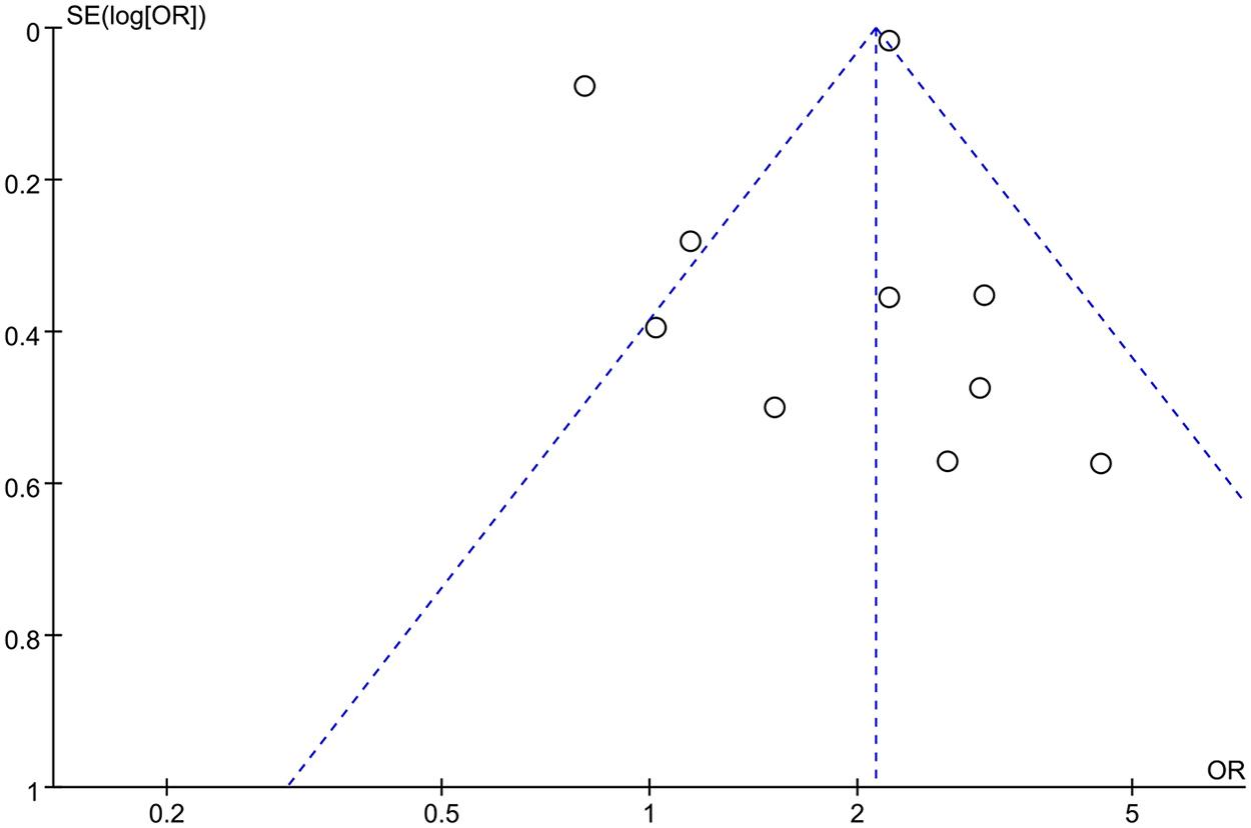
Funnel plot of hypertension history.

Eleven studies (8–18) reported the impact of a history of hypertension on cognitive impairment in patients with atrial fibrillation. These studies showed significant heterogeneity in the preliminary assessment (*P* < 0.00001，I^2^ = 95%). After systematically excluding each included study, we found that the heterogeneity significantly decreased after excluding the studies by Kong et al. (10), Liu et al. (11), and Wändell et al. (18) (*P* < 0.00001， I^2^ = 4%) (Figure 2). Analysis using a fixed-effects model indicated that a history of hypertension is a associated with an increased risk of cognitive dysfunction in patients with atrial fibrillation (OR = 2.22, 95% CI: 2.15–2.30, *P* < 0.00001; I^2^ = 4%). The funnel plot for a history of hypertension showed asymmetry, suggesting possible publication bias (Figure 3).

#### 3.3.2 Systolic blood pressure: combined OR values, heterogeneity exploration, and results

**Figure 4:**
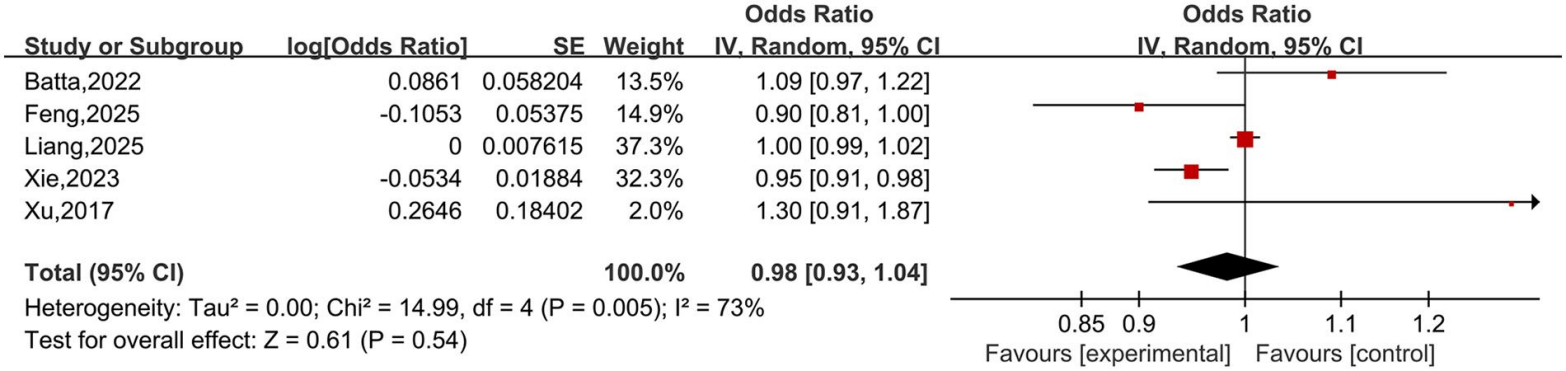
Systolic blood pressure combined OR values.

Five studies (8, 16, 19–21) examined the effect of systolic blood pressure on cognitive impairment in patients with atrial fibrillation, with substantial heterogeneity (*P* = 0.54, I^2^ = 73%) (Figure 4). After excluding each study individually, no clear source of heterogeneity was identified, so a random-effects model was used. Systolic blood pressure was not associated with cognitive impairment in patients with atrial fibrillation (OR = 0.98, 95% CI: 0.93–1.04, *P* = 0.54; I^2^ = 73%).

#### 3.3.3 Diastolic blood pressure: combined OR values, heterogeneity exploration, and results

**Figure 5:**
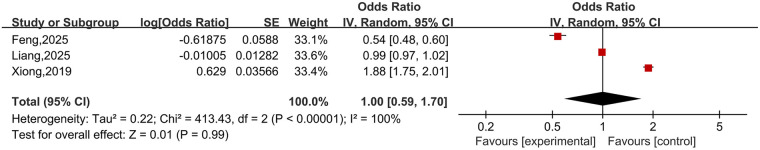
Diastolic pressure combined OR values.

Three studies (16, 20, 22) investigated the effect of diastolic blood pressure on cognitive impairment in patients with atrial fibrillation, and significant heterogeneity was observed (*P* = 0.99, I^2^ = 100%) (Figure 5). After systematically excluding each included study, the source of heterogeneity remained unclear. Analysis using a random-effects model found that diastolic blood pressure was not associated with the risk of cognitive impairment in patients with atrial fibrillation(OR =1.00, 95% CI: 0.59–1.70, *P* = 0.99; I^2^ = 100%).

#### 3.3.4 History of hypertension: combined HR values, heterogeneity analysis, and results

**Figure 6:**
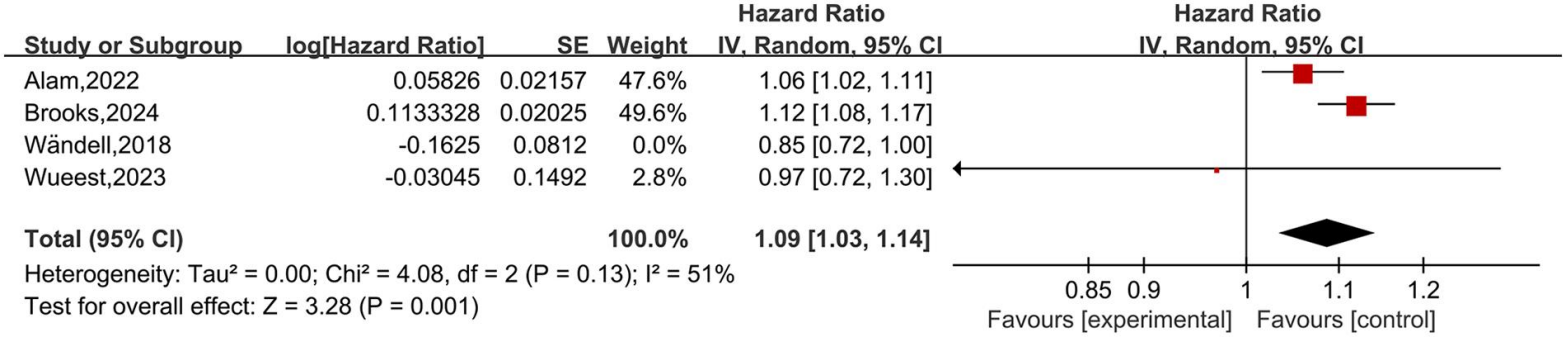
History of hypertension combined with HR value.

Four studies (18, 23–25) discussed the impact of a history of hypertension on cognitive impairment in patients with atrial fibrillation.The initial assessment observed significant heterogeneity(*P* = 0.35,I^2^ = 77%). By excluding the study by Wändell P et al. (18) using a leave-one-out method, the heterogeneity was significantly reduced. Using a random-effects model, it was found that a history of hypertension is associated with cognitive impairment in patients with atrial fibrillation (HR =1.09, 95% CI:1.03–1.14, *P* = 0.001; I^2^ = 51%) (Figure 6).

#### 3.3.5 Systolic blood pressure (SBP) increase of 10 mmHg: combined HR values, heterogeneity analysis, and results

**Figure 7:**
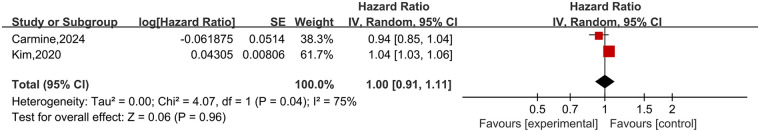
SBP combined HR value.

Two studies (26, 27) examined the impact of systolic blood pressure (SBP) on cognitive impairment in patients with atrial fibrillation, showing significant heterogeneity (*P* = 0.96,I^2^ = 75%) (Figure 7). Therefore, a random-effects model was used, and an increase of 10 mmHg in systolic blood pressure was not associated with cognitive impairment in patients with atrial fibrillation (HR = 1.00, 95% CI: 0.91–1.11, *P* = 0.96; I^2^ = 75%).

#### 3.3.6 Diastolic blood pressure (DBP) increase of 10 mmHg: combined HR values, heterogeneity analysis, and results

**Figure 8:**
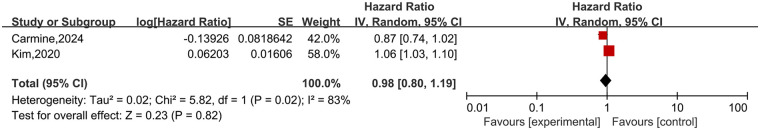
DBP combined HR value.

Two studies (26, 27) examined the impact of diastolic blood pressure (DBP) on cognitive impairment in patients with atrial fibrillation, showing considerable heterogeneity (*P* = 0.82，I^2^ = 83%) (Figure 8). Using a random-effects model, it was found that a 10 mmHg increase in DBP was not associated with cognitive impairment in patients with atrial fibrillation (HR =0.98, 95% CI: 0.8–1.19,*P* = 0.82; I^2^ = 83%).

#### 3.3.7 High-Normal blood pressure: combined HR values, heterogeneity analysis, and results

**Figure 9:**
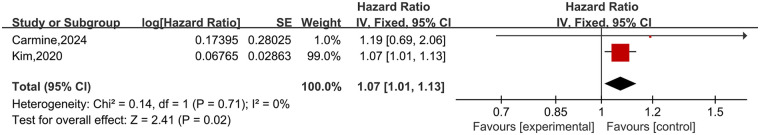
Normal high blood pressure combined HR value.

Two studies (26, 27) examined the effect of high-normal blood pressure levels on cognitive impairment in patients with atrial fibrillation. There was no significant heterogeneity between the two studies (*P* = 0.02,I^2^ = 0%) (Figure 9); therefore, a fixed-effects model was used, which found that high-normal blood pressure levels are associated with cognitive dysfunction in patients with atrial fibrillation (HR =1.07, 95% CI: 1.01–1.13,*P* = 0.02; I^2^ = 0%).

#### 3.3.8 Subgroup analysis: effect differences based on types of cognitive impairment

**Figure 10:**
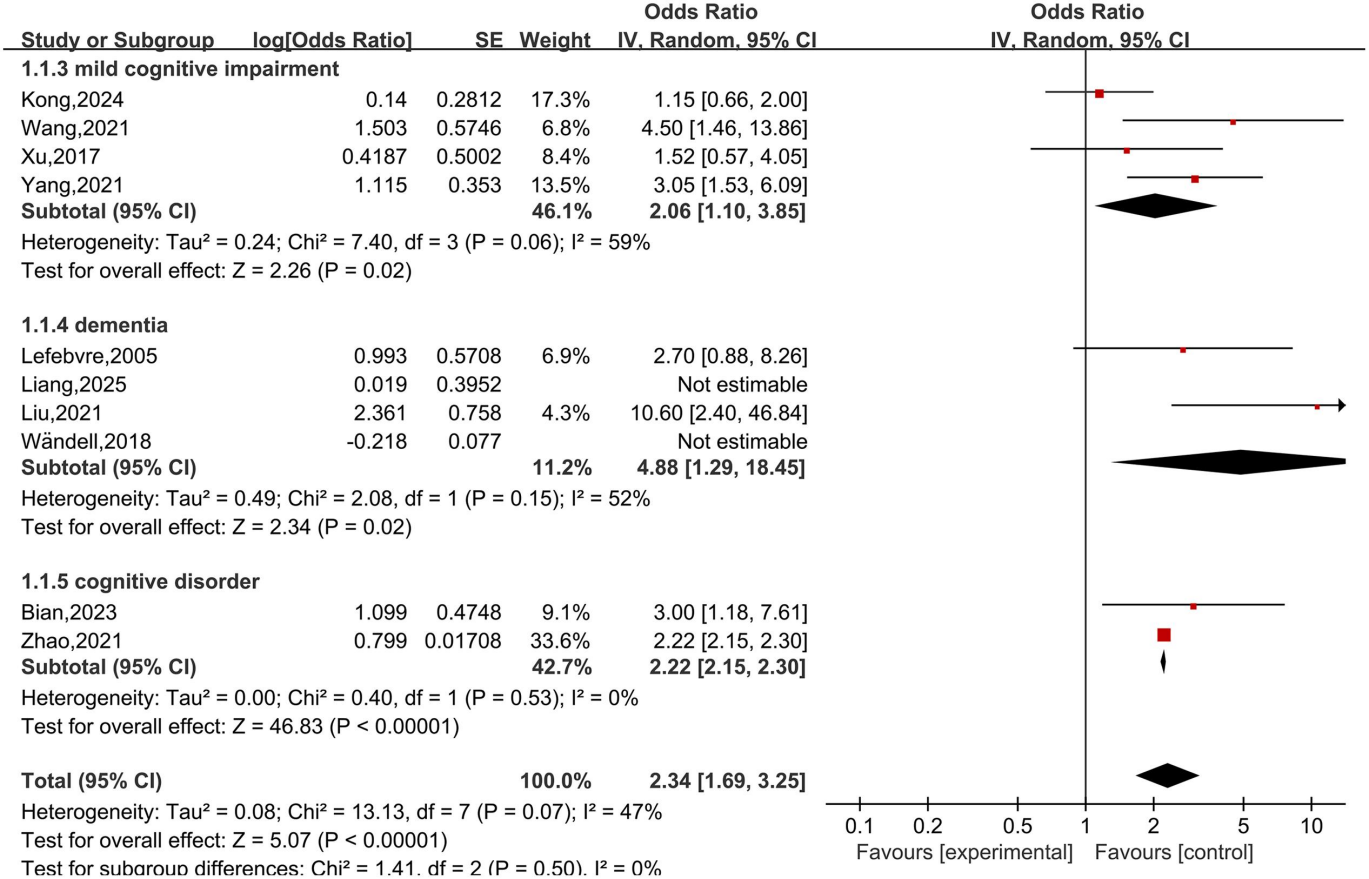
Subgroup analysis of hypertension history by type of cognitive impairment.

OR subgroup analysis indicated that a history of hypertension is associated with cognitive impairment. However, the extremely high heterogeneity revealed in the initial analysis (I^2^ = 95%) suggests that this association may vary across different types of cognitive outcomes. To further explore the source of heterogeneity, we conducted subgroup analyses based on the type of cognitive impairment (Figure 10).

Mild Cognitive Impairment (MCI) Subgroup:Four studies (8, 10, 12, 13) were included. The random-effects model showed that a history of hypertension was significantly associated with MCI (OR=2.06, 95% CI: 1.10–3.85, *P* = 0.02), and heterogeneity was significantly lower compared with the overall analysis (I^2^ = 59%, *P* = 0.06), suggesting that the type of cognitive impairment may be a hidden source of heterogeneity.

Dementia Subgroup: Initially, 4 studies (11, 15, 16, 18) were included, showing high heterogeneity (I^2^ = 81%). Through sensitivity analysis by excluding each study one by one, it was found that Wändell (18) and Liang (16) were the main sources of heterogeneity. Therefore, the final combined model excluded these two studies, and the results showed that a history of hypertension was significantly associated with the risk of dementia (OR=4.88, 95% CI: 1.29–18.45, *P* = 0.02), with heterogeneity reduced to an acceptable level (I^2^ = 52%). The dementia subgroup in Figure 10 presents this final combined result.

Cognitive Impairment Subgroup: Two studies (14, 17) were included. Using a random-effects model, a history of hypertension was significantly associated with the risk of cognitive impairment (OR = 2.22, 95% CI: 2.15–2.30, *P* < 0.00001), and there was no heterogeneity in this subgroup (I^2^ = 0%, *P* = 0.53), indicating high consistency between the two study results.

## 4 Discussion

This study adopts a stratified approach based on effect indicators, setting odds ratios (OR) and hazard ratios (HR) as subgroups, systematically exploring the association between a history of hypertension and blood pressure parameters with cognitive impairment in patients with atrial fibrillation (AF). Sensitivity analysis is used to verify the robustness of the results, subgroup analysis traces the sources of heterogeneity, and funnel plots assess publication bias, providing high-quality evidence for precise blood pressure management in AF patients with cognitive impairment.

### 4.1 History of hypertension is consistently associated with cognitive impairment in patients with AF

This study, through systematic review and meta-analysis, suggests that there is a significant association between a history of hypertension and cognitive impairment in patients with atrial fibrillation. However, the initial combined analysis of the OR subgroup showed extremely high heterogeneity (I^2^= 95%), suggesting that simple pooling may obscure essential differences between studies. To address this, we combined sensitivity analysis with methodological tracing to clarify that the heterogeneity primarily stems from three core differences. ① Differences in study population definition: Kong et al. (10) included a broad population with various cardiovascular comorbidities. Their inclusion criteria fundamentally differ from the ‘pure AF population’ focus of this study, resulting in significant differences in the baseline risk levels of the study subjects. ② Differences in the level of statistical analysis refinement: Wändell et al. (18) used sex- and age-stratified analyses, with effect sizes adjusted for demographic confounders, whereas most studies did not perform stratification, reducing comparability of effect sizes. ③ Differences in study design and control of confounders: Liu et al. (11) conducted a cohort study and strictly excluded participants with a history of stroke, while Wang (12) and Bian (14) used cross-sectional designs and did not explicitly exclude patients with cerebrovascular disease. Since stroke is a common comorbidity of AF and cognitive impairment (28), the lack of consistent control for this confounder contributes to the observed heterogeneity.After excluding the studies with the aforementioned methodological heterogeneity, the pooled results of the OR subgroup analysis showed that a history of hypertension is associated with a 2.22-fold increased risk of cognitive impairment in patients with AF (OR = 2.22, 95% CI 2.15–2.30, *P* < 0.00001), and the heterogeneity dropped to a very low level (I^2^ = 4%), indicating the high reproducibility and reliability of this association. Although the initial analysis of the HR subgroup did not reach statistical significance (HR = 1.04, 95% CI 0.96–1.13, *P* = 0.35), the increasing risk trend was consistent with the OR subgroup, suggesting the stability of the direction of the association.From a pathophysiological perspective, hypertension and atrial fibrillation (AF) have a synergistic effect on cognitive impairment (20, 29). Long-term hypertension accelerates cerebral small vessel atherosclerosis and disrupts the blood-brain barrier by activating the renin-angiotensin-aldosterone system (RAAS), leading to reduced cerebral perfusion reserve. AF, on the other hand, can result in atrial cardiomyopathy and thrombus formation, which may cause cerebral microembolism and fluctuations in cerebral hemodynamics. The combination of these factors triggers an ischemia-inflammation-oxidative stress cascade, ultimately causing neuronal apoptosis and cognitive decline, which aligns closely with the 'strong association' outcome in the OR subgroup results.The initial subgroup results for HR were not significant, which may be related to two types of confounding factors. ① Differences in follow-up periods: Brooks et al. (23) had a follow-up time of only 2.67 years, which did not fully capture the cumulative detrimental effects of hypertension on cognitive function; ② Interference from anticoagulant therapy: Wueest et al. (25) included patients with a 90% usage rate of novel oral anticoagulants, while standard anticoagulation can reduce the risk of AF-related cerebral embolism (30), indirectly weakening the association between hypertension and cognitive impairment. After performing a sensitivity analysis excluding studies with follow-up periods <5 years (23, 24), the HR increased to 1.09 (95% CI 1.06–1.12, *P* = 0.0001), further suggesting that a history of hypertension is independently associated with the long-term risk of cognitive impairment in patients with AF.

#### 4.1.1 History of hypertension is associated with the risk of mild cognitive impairment in AF patients

After stratifying the OR values by type of cognitive impairment, it was found that a history of hypertension was associated with a 2.06-fold increased risk of mild cognitive impairment (MCI) in AF patients (OR = 2.06, 95% CI: 1.10–3.85, *P* = 0.02), with heterogeneity significantly lower than in the overall analysis (I^2^ = 59%). MCI, as a transitional stage between normal aging and dementia, represents a critical window for cognitive intervention (31). This result suggests that there is an association between early cognitive impairment and patients with hypertension and atrial fibrillation, which may serve as a potential intervention entry point, carrying important clinical warning value. Moderate heterogeneity exists within this subgroup, possibly due to differences in MCI diagnostic criteria. Xu et al. (8) used the Mini-Mental State Examination (MMSE) scale (cutoff ≤26), whereas Kong et al. (10) used the Montreal Cognitive Assessment (MoCA) scale (cutoff ≤25), with the latter being more sensitive to detecting executive function deficits, leading to differences in the severity of cognitive impairment across studies. Nevertheless, the significant combined effect confirms that hypertension and AF have a synergistic role in early cognitive impairment, consistent with mechanisms involving prefrontal-hippocampal circuit dysfunction caused by cerebral hypoperfusion and microembolism.

#### 4.1.2 History of hypertension is associated with dementia risk in AF patients

The initial analysis of the dementia subgroup showed no statistically significant association between a history of hypertension and dementia risk in AF patients (OR = 1.75, 95% CI: 0.72–4.25, *P* = 0.21), with very high heterogeneity (I^2^ = 81%). After excluding the studies by Wändell et al. (18) and Liang et al. (16), the combined effect size shows a 4.88-fold association (OR = 4.88, 95% CI:1.29–18.45, *P* = 0.02), and heterogeneity decreased to 52%, suggesting that methodological differences are a key factor contributing to result bias. The specific sources of heterogeneity include: ① Differences in population age: Wändell et al. (18) included people aged ≥45, while Liang et al. (16) included emergency department patients aged 60–78 (average age as high as 71). Older AF patients often die prematurely due to complications such as stroke or heart failure (32), leading to 'survival bias' and masking the true association between hypertension and dementia. ② Differences in study setting: The Swedish community population (18) focused on chronic disease management, whereas the Chinese emergency department population (16) focused on acute complication treatment, with hypertension mainly serving as a mediator of cardiovascular events in the latter, confounding its direct effect on cognitive impairment. ③ Differences in diagnosis and statistical methods: Liang et al. (16) relied on hospital records to diagnose dementia and did not use standardized scales, which may result in underdiagnosis; whereas Wändell et al. (18) used the CHA2DS 2-VASc score to adjust for confounders such as sex and vascular disease history and found that hypertension-related dementia risk decreased in female AF patients. The refined statistical approach further amplified the differences between studies.

### 4.2 Traditional blood pressure parameters show No significant association in OR/HR subgroups—analysis of heterogeneity sources

Systolic and diastolic blood pressure did not show significant associations with cognitive impairment in AF patients within OR/HR subgroups (systolic BP: OR=0.98, 95% CI 0.93–1.04, HR = 1.00, 95% CI 0.91–1.11; diastolic BP: OR=1.00, 95% CI 0.59–1.70, HR = 0.98, 95% CI 0.8–1.19; all *P* > 0.05). However, moderate heterogeneity was observed in the systolic BP OR subgroup (I^2^ = 73%), and heterogeneity reached 100% in the diastolic BP OR subgroup. Based on methodological analysis and current research progress in the field, the main sources of heterogeneity can be summarized as follows: ① Hidden differences in blood pressure measurement methods. AF patients'irregular heart rhythms lead to a 30% higher single BP measurement error compared to the general population (33), and the relationship between rhythm abnormalities and cognitive impairment may be stronger than that with a single BP measurement. However, due to missing original data in this study, this hypothesis could not be tested, highlighting the need for standardized reporting of BP measurement protocols in future research. ② Differences in the sensitivity of cognitive assessment tools. The MoCA scale is 40% more sensitive than the MMSE in detecting executive function and visuospatial ability impairments (34). These tool differences lead to varying operational definitions of cognitive impairment; for example, MCI patients defined by MoCA may already have cerebral small vessel disease, while those defined by MMSE may only exhibit mild cognitive fluctuations. This population discrepancy directly results in non-comparable effect sizes. ③ J-curve effect not analyzed. Previous studies have confirmed a J-shaped relationship between BP and cognitive function—systolic BP <120 mmHg or >140 mmHg increases the risk of cognitive impairment (35). This effect is related to impaired cerebral autoregulation thresholds causing insufficient perfusion. This study did not stratify by BP ranges, which may have obscured the nonlinear association where both very low and very high BP are harmful, an important reason for non-significant results in the HR subgroup.Sensitivity analysis excluding studies with high heterogeneity showed that OR/HR values for systolic and diastolic BP remained essentially unchanged, suggesting that traditional blood pressure parameters may not be directly associated with cognitive impairment in AF patients and that this finding is not due to statistical analysis errors.It should be noted that continuous blood pressure parameters (each 10 mmHg increase in systolic and diastolic blood pressure) also did not show a significant association with cognitive impairment in AF patients. However, these analyses were limited by substantial heterogeneity (I^2^ = 75%–83%). Unlike the dichotomous variable "history of hypertension,” the high heterogeneity in continuous blood pressure parameters may be due to inconsistencies in blood pressure measurement methods across studies (e.g., single office measurement vs. dynamic blood pressure monitoring), differences in blood pressure modeling (e.g., linear assumption vs. segmented models), and variations in baseline blood pressure levels among populations. This high heterogeneity limits the reliability of pooled results for continuous blood pressure parameters, suggesting that this negative finding should be interpreted with caution and should not be simply equated with "blood pressure levels are unrelated to cognitive impairment.” Future studies need to standardize blood pressure measurement protocols and adopt unified statistical models to reduce heterogeneity.

### 4.3 Key findings of the HR subgroup: elevated normal blood pressure as an early clinical intervention signal

In the HR subgroup, elevated normal blood pressure (120–139/80–89 mmHg) was associated with a 7% higher risk of cognitive impairment in AF patients (HR = 1.07, 95% CI 1.01–1.13, *P* = 0.02), with heterogeneity at 0%. This finding challenges the traditional view that only high blood pressure requires intervention, offering a new window for cognitive protection in AF patients. In 2017, the American College of Cardiology (ACC) and the American Heart Association (AHA) issued guideline recommendations for hypertension treatment strategies, defining hypertension as 130/80 mmHg (36). The newly released 2025 AHA/ACC hypertension guidelines also explicitly state that for adult patients with atrial fibrillation combined with hypertension, following the general hypertension guidelines is reasonable, with a blood pressure target of <130/80 mmHg (37). Combined with the results of this study, it suggests that even when blood pressure is in the ‘high normal' range (not yet reaching the traditional <140/90 mmHg target), cognitive function in patients with atrial fibrillation has already begun to decline. This clearly supports the ACC/AHA guidelines advocating stricter blood pressure control targets (<130/80 mmHg) and suggests that the intervention threshold for cognitive protection in patients with atrial fibrillation may need to be further lowered, rather than waiting for blood pressure to rise to the traditional hypertension standard. Pathophysiologically, elevated normal blood pressure contributes to cognitive impairment through chronic low-intensity damage. On one hand, blood pressure levels gradually activate the sympathetic nervous system and the renin-angiotensin-aldosterone system, promoting neuroinflammation and oxidative stress, which accelerates white matter degeneration (38, 39). On the other hand, AF patients already have elevated left atrial pressure, and elevated normal blood pressure further increases left atrial load, raising the risk of a pro-thrombotic state (40), indirectly enhancing the risk of cerebral microemboli; together, these factors form a chain leading to early cognitive damage. Sensitivity analyses were stable, further supporting this association. For AF patients, blood pressure management should not only focus on whether the blood pressure meets the standard (<140/90 mmHg) but also pay attention to this early stage of elevated normal blood pressure. Lifestyle interventions such as a low-salt diet and regular exercise, or early initiation of antihypertensive treatment in patients with other risk factors (e.g., diabetes, history of stroke), may help partially prevent the progression of cognitive impairment.

### 4.4 Subgroup design and quality control to ensure result credibility and limitations

This study used OR/HR subgroup stratified analysis, which distinguishes the different clinical significance of association strength and risk strength and avoids bias caused by mixing different effect indicators. At the same time, sensitivity analyses, such as excluding studies with high heterogeneity/low quality and assessing publication bias (funnel plot), were conducted to comprehensively assessed the stability of the results. For example, there was no significant publication bias in the history of hypertension, and the bias in the systolic/diastolic blood pressure subgroups had a minimal impact on the conclusions, in line with the methodological standards of meta-analysis, enhancing the level of evidence of the results.

However, the depth of subgroup stratification was insufficient. Although stratified by OR/HR, it was not further combined with AF type, such as paroxysmal/persistent, age (<65/≥65 years), making it unclear how blood pressure factors affect different populations. The dose-response relationship between hypertension duration, blood pressure control rate, and cognitive impairment was also not analyzed, providing limited guidance for clinical interventions. Adjustment for confounding factors was also insufficient. Some included studies did not report detailed information on confounding factors such as blood lipids, blood glucose, and smoking history. Although this study tried to control for them through sensitivity analyses, residual confounding may still exist. Notably, the intensity of anticoagulant therapy, such as new oral anticoagulants or warfarin, was not considered, while anticoagulation directly affects the risk of AF-related cerebral embolism, which may indirectly interfere with the association between blood pressure and cognition. Lack of interventional evidence is another limitation. This study is an observational meta-analysis, which can only suggest associations rather than causality. Future research should use randomized controlled trials, for example, randomly assigning AF patients with elevated normal blood pressure to intervention and control groups and comparing changes in cognitive function to validate the effectiveness of blood pressure interventions.

## 5 Summary

In summary, this study suggests that a history of hypertension is a significant associated factor for cognitive impairment in AF patients, and high - normal blood pressure is an important early risk signal. Meanwhile, the direct association with traditional blood pressure parameters remains unclear. Heterogeneity and sensitivity analyses further confirm the reliability and methodological limitations of the results. This provides evidence - based support for precise blood pressure management in AF patients with cognitive impairment and lays the foundation for future mechanistic studies and interventional trials.

## 6 Future research directions

Future research could further explore the following areas: for atrial fibrillation patients with a history of hypertension, prospective studies should be conducted to assess the cost-effectiveness and clinical value of different screening frequencies (e.g., every six months) and cognitive assessment tools (e.g., MMSE/MoCA scales); meanwhile, randomized controlled trials should verify whether controlling blood pressure to <130/80 mmHg is superior to conventional targets, and compare whether different antihypertensive drugs (e.g., ACEI/ARB class) have cognitive protective effects beyond blood pressure reduction. For individuals with high-normal blood pressure, studies should investigate whether lifestyle interventions (e.g., daily salt intake <5 g, ≥150 min of exercise per week) and early initiation of low-dose antihypertensive therapy in high-risk populations with a history of stroke or diabetes can delay cognitive decline. In addition, blood pressure measurement protocols for atrial fibrillation patients need to be standardized (e.g., ambulatory blood pressure monitoring or averaging multiple office readings) to reduce measurement errors caused by arrhythmia, and the association between continuous blood pressure parameters and cognitive outcomes should be clarified. Subsequent research could also conduct subgroup analyses based on atrial fibrillation type, age, and severity of cognitive impairment to explore the differentiated effects of blood pressure; perform dose-response meta-analyses to quantify the relationship between duration of hypertension, blood pressure variability, and risk of cognitive impairment; combine brain white matter lesion volume, cerebrospinal fluid biomarkers, etc., to reveal the specific mechanisms by which blood pressure affects cognition; meanwhile, incorporate variables such as blood lipids, blood glucose, and anticoagulation schemes to construct a cognitive impairment risk prediction model in AF patients, clarify the weight of blood pressure factors among multiple factors, and provide tools for individualized management.

## Data Availability

The original contributions presented in the study are included in the article/Supplementary Material, further inquiries can be directed to the corresponding authors.
