# Supplementary material for: Impact of hypertension history and blood pressure parameters on cognitive impairment in patients with atrial fibrillation: a systematic review and meta-analysis
Source: Front Cardiovasc Med. 2026 Jun 2;13:1820778. doi: 10.3389/fcvm.2026.1820778 (PMC13268889; doi:10.3389/fcvm.2026.1820778)
Supplement: Supplementary file 1 [file Datasheet1.docx]

**Table S1 Search strategy**

| Databases | Search strategy |
| --- | --- |
| PubMed | #5 (#1 OR #3) AND (#2 OR #4)  #4 (((((((((((((((((((((((((((((((((((Cognitive Dysfunction[Title/Abstract]) OR (Cognitive Impairment[Title/Abstract])) OR (mild cognitive impairment[Title/Abstract])) OR (MCI[Title/Abstract])) OR (Cognitive Decline[Title/Abstract])) OR (MCI, Dementia[Title/Abstract])) OR (Alzheimer Disease[Title/Abstract])) OR (Cognition[Title/Abstract])) OR (Amentia[Title/Abstract])) OR (Senile Paranoid Dementia[Title/Abstract])) OR (Familial Dementia[Title/Abstract])) OR (Mental Deterioration[Title/Abstract])) OR (Deteriorations, Mental[Title/Abstract])) OR (Mental Deteriorations[Title/Abstract])) OR (Dementias, Vascular[Title/Abstract])) OR (Arteriosclerotic Dementia[Title/Abstract])) OR (Encephalopathy, Binswanger[Title/Abstract])) OR (Chronic Progressive Subcortical Encephalopathy[Title/Abstract])) OR (Leukoencephalopathy, Subcortical[Title/Abstract])) OR (Encephalopathy, Subcortical Arteriosclerotic[Title/Abstract])) OR (Binswanger's Encephalopathy[Title/Abstract])) OR (Binswanger's Disease[Title/Abstract])) OR (Vascular Dementias, Subcortical[Title/Abstract])) OR (Acute Onset Vascular Dementia[Title/Abstract])) OR (Diffuse Lewy Body Disease[Title/Abstract])) OR (Lewy Body Disease, Cortical[Title/Abstract])) OR (Lewy Body Dementia[Title/Abstract])) OR (Dementias, Frontotemporal[Title/Abstract])) OR (Frontotemporal Lobe Dementias [Title/Abstract]))) OR (Dementia, Ubiquitin-Positive Frontotemporal[Title/Abstract])) OR (Disorder, Neurocognitive[Title/Abstract])) OR (Mental Disorders, Organic[Title/Abstract])) OR (Psychoses, Traumatic[Title/Abstract])) OR (Mild Neurocognitive Disorder[Title/Abstract])) OR (Organic Mental Disorder[Title/Abstract])) OR (Organic Mental Disorders, Psychotic[Title/Abstract])  #3 ((((((Atrial Fibrillation[Title/Abstract]) OR (atrial fibrillat*[Title/Abstract])) OR (Atrial Flutter[Title/Abstract])) OR (AFib[Title/Abstract])) OR (AF[Title/Abstract])) OR (auricular fibrillat*[Title/Abstract]))  #2 ((((("Cognitive Dysfunction"[Mesh]) OR ("Dementia, Vascular"[Mesh])) OR ("Lewy Body Disease"[Mesh])) OR ("Neurocognitive Disorders"[Mesh])) OR ("Frontotemporal Dementia"[Mesh])) OR ("Alzheimer Disease"[Mesh])  #1 "Atrial Fibrillation"[Mesh] |
|  | #7 #5 AND #6 |
| EMBASE | #6 3 OR #4  #5 #1 OR #2  #4 'Atrial Fibrillation':ti,ab,kw OR 'atrial fibrillat*':ti,ab,kw OR 'Atrial Flutter':ti,ab,kwOR 'AFib':ti,ab,kw OR 'AF':ti,ab,kw OR 'auricular fibrillat*':ti,ab,kw  #3 'Atrial Fibrillation'/exp  #2 dementia:ti,ab,kw OR demention:ti,ab,kw OR 'cognition disorder':ti,ab,kw OR 'cognitive complaints':ti,ab,kw OR 'cognitive defect':ti,ab,kw OR 'cognitive dysfunction':ti,ab,kw OR 'cognitive impairment':ti,ab,kw OR 'cognitive problems':ti,ab,kw OR 'delirium, dementia, amnestic, cognitive disorders':ti,ab,kw OR 'alzeimer diseas':ti,ab,kw OR 'alzheimer dementia':ti,ab,kw OR 'alzheimer fibrillary change':ti,ab,kw OR 'alzheimer syndrome':ti,ab,kw OR 'dementia, multiinfarct':ti,ab,kw OR 'dementia, vascular':ti,ab,kw OR 'lacunar dementia':ti,ab,kw OR 'multi-infarct dementia':ti,ab,kw OR 'lewy body dementia':ti,ab,kw OR 'diffuse lewy body disease':ti,ab,kw OR 'dementia with lewy bodies':ti,ab,kw OR 'neuro-cognitive disorder':ti,ab,kw OR 'neurocognitive dysfunction':ti,ab,kw OR 'disorders of higher cerebral function':ti,ab,kw OR 'neurocognitive syndrome':ti,ab,kw OR 'frontotemporal dementia':ti,ab,kw OR 'dementia, frontotemporal':ti,ab,kw OR 'frontotemporal dementias':ti,ab,kw  #1 'Cognitive Dysfunction'/exp OR 'Cognitive defect'/exp OR 'Alzheimer Disease'/exp OR 'Lewy Body Disease'/exp OR 'Frontotemporal Dementia'/exp OR 'Dementia, Vascular'/exp |
| The Cochrane Library | #1 (MCI ):ti,ab,kw OR (mild cognitive impairment):ti,ab,kw OR ( Cognitive Dysfunction):ti,ab,kw OR ( Cognitive Impairment):ti,ab,kw OR (Cognitive Decline):ti,ab,kw OR (Neurocognitive Disorders ):ti,ab,kw OR ( Dementia ):ti,ab,kw OR (Alzheimer Disease):ti,ab,kw OR (Cognition):ti,ab,kw OR (Cognitive Disorder):ti,ab,kw OR ( Transmissible Dementia ):ti,ab,kw OR (Dementia, Hereditary Multi-Infarct Type):ti,ab,kw OR ( Dementia Praecox ):ti,ab,kw OR ( Dementia, Lewy Body):ti,ab,kw OR (Dementia, HIV ):ti,ab,kw OR ( Acute Onset Vascular Dementia):ti,ab,kw OR ( Vascular Dementia ):ti,ab,kw OR ( Alzheimer Dementias ):ti,ab,kw OR ( Frontotemporal Dementias ):ti,ab,kw OR ( Dementia Multi Infarct):ti,ab,kw  #2 (Atrial Fibrillation):ti,ab,kw OR (atrial fibrillat):ti,ab,kw OR (Atrial Flutter):ti,ab,kw OR ( AFib ):ti,ab,kwOR ( AF):ti,ab,kw  #3 #1 AND #2 |
| Web of science | #1 (((((((TS=(MCI) OR TS=( mild cognitive impairment )) OR TS=( Cognitive Dysfunction )) OR TS=( Cognitive Impairment)) OR TS=(Cognitive Decline)) OR TS=(Neurocognitive Disorders)) OR TS=(Dementia)) OR TS=(Alzheimer Disease )) OR TS=( Cognition ) OR TS=( MoCA) OR TS=(Dementia Multi Infarct) OR TS=(Frontotemporal Dementias ) OR TS=(Alzheimer Dementias ) OR TS=(Dementia, Hereditary Multi-Infarct Type) OR TS=(Vascular Dementia )  #2 ((TS=(Atrial Fibrillation ) OR TS=(atrial fibrillat)) OR TS=(Atrial Flutter )) OR TS=( AFib ) OR TS=( AF )  #3 #2 AND #1 |
| CNKI | #1（主题：心房颤动） OR （主题：房颤） OR （主题：耳颤动） OR （主题：持续性心房颤动） OR （主题：家族性心房颤动） OR （主题：家族性心房颤动）OR （主题：阵发性心房颤动）  #2（主题：认知功能障碍） OR （主题：痴呆） OR （主题：认知障碍） OR （主题：轻度认知障碍） OR （主题：认知能力下降） OR （主题：痴呆）OR （主题：家族性痴呆）OR （主题：阿尔茨海默病） OR （主题：认知功能）OR （主题：MMSE）OR (主题：多发梗死性痴呆) OR (主题：额颞叶痴呆) OR (主题：神经认知障碍) OR (主题：神经认知综合征) OR (主题：高级脑功能障碍) OR (主题：路易体痴呆) OR(主题：路易体痴呆症群) OR (主题：多发性梗死性痴呆) OR (主题：血管性痴呆) OR (主题：腔隙性痴呆) OR (主题：阿尔茨海默硬化症) OR (主题：认知缺陷) OR (主题：认知衰弱) OR (主题：认知不足) OR (主题：认知障碍症) OR (主题：认知衰退)  #3 #1 AND #2 |
| Wanfang | 主题:(心房颤动 OR 房颤 OR 耳颤动 OR 持续性心房颤动OR 家族性心房颤动OR 阵发性心房颤动) AND 主题:(认知功能障碍OR痴呆OR 认知障碍OR 轻度认知障碍OR 认知能力下降OR 痴呆OR 家族性痴呆OR 阿尔茨海默病OR 认知功能OR认知损害OR MMSE OR MoCA OR 认知衰退 OR 认知衰弱 OR 认知缺陷 OR 认知障碍症 OR 腔隙性痴呆 OR 神经认知障碍 OR 血管性痴呆 OR 路易体痴呆) |
| CBM | #1认知障碍[不加权：扩展]  #2认知功能障碍OR痴呆OR 认知障碍OR 轻度认知障碍OR 认知能力下降OR 痴呆OR 家族性痴呆OR 阿尔茨海默病OR 认知功能OR认知损害OR MMSE OR MoCA OR 认知衰弱OR 认知障碍症 OR 认知损伤 OR 血管性痴呆 OR 腔隙性痴呆OR 认知衰退 OR 阿尔茨海默病 OR 阿尔茨海默痴呆 OR 多发性梗死性痴呆 OR 路易体痴呆症群 OR 神经认知症状 OR 额颞叶痴呆  #3 #1 OR #2  #4 心房颤动[不加权：扩展]  #5心房颤动 OR 房颤 OR 耳颤动 OR 持续性心房颤动OR 家族性心房颤动OR 阵发性心房颤动  #6 #4 OR #5  # #3 AND#6 |
| VIP | M=(心房颤动 OR 房颤 OR 耳颤动 OR 持续性心房颤动OR 家族性心房颤动OR 阵发性心房颤动  ) AND M=(认知功能障碍OR痴呆OR 认知障碍OR 轻度认知障碍OR 认知能力下降OR 痴呆OR 家族性痴呆OR 阿尔茨海默病OR 认知功能OR认知损害OR MMSE OR MoCA OR 认知衰弱OR 认知障碍症 OR 认知损伤 OR 血管性痴呆 OR 腔隙性痴呆OR 认知衰退 OR 阿尔茨海默病 OR 阿尔茨海默痴呆 OR 多发性梗死性痴呆 OR 路易体痴呆症群 OR 神经认知症状 OR 额颞叶痴呆) |
