# Supplementary material for: Impact of hypertension history and blood pressure parameters on cognitive impairment in patients with atrial fibrillation: a systematic review and meta-analysis
Source: Front Cardiovasc Med. 2026 Jun 2;13:1820778. doi: 10.3389/fcvm.2026.1820778 (PMC13268889; doi:10.3389/fcvm.2026.1820778)
Supplement: Supplementary file 2 [file Datasheet2.docx]

| **Section and Topic** | **Item #** | **Checklist item** | **Location where item is reported** |
| --- | --- | --- | --- |
| **TITLE** | | |  |
| Title | 1 | Identify the report as a systematic review. | Title:（Impact of Hypertension History and Blood Pressure Parameters on Cognitive Impairment in Patients with Atrial Fibrillation: A Systematic Review and Meta-Analysis） |
| **ABSTRACT** | | |  |
| Abstract | 2 | See the PRISMA 2020 for Abstracts checklist. | Abstract (structured abstract including objectives, methods, results, and conclusions) |
| **INTRODUCTION** | | |  |
| Rationale | 3 | Describe the rationale for the review in the context of existing knowledge. | Introduction paragraph (describes the relationship between atrial fibrillation and cognitive impairment, the role of  hypertension, and the limitations of existing research) |
| Objectives | 4 | Provide an explicit statement of the objective(s) or question(s) the review addresses. | The final paragraph of the introduction ('the present study aims to adopt a stratified analysis approach to systematically evaluate...') |
| **METHODS** | | |  |
| Eligibility criteria | 5 | Specify the inclusion and exclusion criteria for the review and how studies were grouped for the syntheses. | Section 1.2 (details the inclusion and exclusion criteria) |
| Information sources | 6 | Specify all databases, registers, websites, organisations, reference lists and other sources searched or consulted to identify studies. Specify the date when each source was last searched or consulted. | Section 1.1 (Lists 8 databases, search time up to September 7, 2025) |
| Search strategy | 7 | Present the full search strategies for all databases, registers and websites, including any filters and limits used. | Section 1.1 (Describes Chinese and English search terms, mentioning that the detailed PubMed strategy is in Figure 1) |
| Selection process | 8 | Specify the methods used to decide whether a study met the inclusion criteria of the review, including how many reviewers screened each record and each report retrieved, whether they worked independently, and if applicable, details of automation tools used in the process. | Section 1.3 (describes two researchers independently screening the literature and using EndNote 21 to remove duplicates) |
| Data collection process | 9 | Specify the methods used to collect data from reports, including how many reviewers collected data from each report, whether they worked independently, any processes for obtaining or confirming data from study investigators, and if applicable, details of automation tools used in the process. | Section 1.3 (describes how two researchers independently extracted data and cross-checked it) |
| Data items | 10a | List and define all outcomes for which data were sought. Specify whether all results that were compatible with each outcome domain in each study were sought (e.g. for all measures, time points, analyses), and if not, the methods used to decide which results to collect. | Section 1.2.1(5) (Cognitive impairment, dementia, or mild cognitive impairment); Section 1.2.1(4) (Studies providing adjusted OR values, HR values, and 95% CI) |
|  | 10b | List and define all other variables for which data were sought (e.g. participant and intervention characteristics, funding sources). Describe any assumptions made about any missing or unclear information. | Section 1.3 (extracted information such as first author, year of publication, country, study design, sample size, specific exposure variables, etc.); Section 1.4 (did not make assumptions about missing or unclear information, evaluated using quality assessment tools) |
| Study risk of bias assessment | 11 | Specify the methods used to assess risk of bias in the included studies, including details of the tool(s) used, how many reviewers assessed each study and whether they worked independently, and if applicable, details of automation tools used in the process. | Section 1.4 (Provides a detailed description of using the NOS scale to assess cohort and case-control studies, and the AHRQ tool to assess cross-sectional studies; two researchers conducted the assessments independently) |
| Effect measures | 12 | Specify for each outcome the effect measure(s) (e.g. risk ratio, mean difference) used in the synthesis or presentation of results. | Section 1.5 (Using OR values, HR values, and their 95% confidence intervals) |
| Synthesis methods | 13a | Describe the processes used to decide which studies were eligible for each synthesis (e.g. tabulating the study intervention characteristics and comparing against the planned groups for each synthesis (item #5)). | Section 1.5 ('Considering the clinical heterogeneity of OR and HR, they were combined separately'); Section 2.3 ('Six risk factors were extracted from 20 studies, and a meta-analysis was conducted on three of them…') |
|  | 13b | Describe any methods required to prepare the data for presentation or synthesis, such as handling of missing summary statistics, or data conversions. | Section 1.5 (Methods for handling missing data were not specifically described, but all included studies provided OR/HR and 95% CI) |
|  | 13c | Describe any methods used to tabulate or visually display results of individual studies and syntheses. | Table 1 (Summary of basic information of included studies); Figure 2 (Literature screening process); Figures 3, 5, 6, 7, 8, 9, 10, 11 (Forest plots showing Meta-analysis results); Figure 4 (Funnel plot) |
|  | 13d | Describe any methods used to synthesize results and provide a rationale for the choice(s). If meta-analysis was performed, describe the model(s), method(s) to identify the presence and extent of statistical heterogeneity, and software package(s) used. | Section 1.5 (Provides a detailed description of using RevMan 5.4 software; employing either a fixed-effect model or a random-effect model; using the I² statistic to assess heterogeneity) |
|  | 13e | Describe any methods used to explore possible causes of heterogeneity among study results (e.g. subgroup analysis, meta-regression). | Section 1.5 ("We also conducted exploratory subgroup analyses"); Sections 2.3.1, 2.3.4, etc. (exploring heterogeneity by excluding studies one by one); Section 2.3.8 (subgroup analysis based on types of cognitive impairment) |
|  | 13f | Describe any sensitivity analyses conducted to assess robustness of the synthesized results. | Section 1.5 ("Conduct sensitivity analysis to verify the stability of results"); Sections 2.3.1, 2.3.4, 2.3.8, etc. (describe in detail the process and results of the sensitivity analysis) |
| Reporting bias assessment | 14 | Describe any methods used to assess risk of bias due to missing results in a synthesis (arising from reporting biases). | Section 1.5 ("For meta-analyses including ten or more studies, publication bias is assessed by evaluating the symmetry of funnel plots"); Section 2.3.1 (Publication bias was assessed for a history of hypertension) |
| Certainty assessment | 15 | Describe any methods used to assess certainty (or confidence) in the body of evidence for an outcome. | Not explicitly described (this article did not use methods such as GRADE to assess the certainty of the evidence) |
| **RESULTS** | | |  |
| Study selection | 16a | Describe the results of the search and selection process, from the number of records identified in the search to the number of studies included in the review, ideally using a flow diagram. | Section 2.1 (describes the process and specific numbers of retrieval, deduplication, screening, and final inclusion); Figure 2 (PRISMA flow diagram) |
|  | 16b | Cite studies that might appear to meet the inclusion criteria, but which were excluded, and explain why they were excluded. | The exclusion criteria are clearly stated in Section 1.2.2. They are usually listed in the PRISMA flow diagram as 'Excluded full-text articles and reasons. |
| Study characteristics | 17 | Cite each included study and present its characteristics. | Section 2.2 (describes the basic characteristics of the included studies); Table 1 (summarizes the basic information of the included studies) |
| Risk of bias in studies | 18 | Present assessments of risk of bias for each included study. | Section 2.2 (Describes the results of the quality assessment: "All included cross-sectional studies had AHRQ scores of ≥4, and all included cohort and case-control studies had NOS scores of ≥6, indicating that the study quality was relatively high") |
| Results of individual studies | 19 | For all outcomes, present, for each study: (a) summary statistics for each group (where appropriate) and (b) an effect estimate and its precision (e.g. confidence/credible interval), ideally using structured tables or plots. | Figures 3, 5, 6, 7, 8, 9, 10, 11 (The forest plots show the effect estimates of individual studies and their confidence intervals) |
| Results of syntheses | 20a | For each synthesis, briefly summarise the characteristics and risk of bias among contributing studies. | Implicitly described in each meta-analysis results section, but not systematically summarized |
|  | 20b | Present results of all statistical syntheses conducted. If meta-analysis was done, present for each the summary estimate and its precision (e.g. confidence/credible interval) and measures of statistical heterogeneity. If comparing groups, describe the direction of the effect. | Sections 2.3.1 to 2.3.8 (detailed reports of the combined effect sizes, confidence intervals, and heterogeneity indicators of each meta-analysis) |
|  | 20c | Present results of all investigations of possible causes of heterogeneity among study results. | Sections 2.3.1, 2.3.4, 2.3.8, etc. (exploring the sources of heterogeneity through sensitivity analysis and subgroup analysis) |
|  | 20d | Present results of all sensitivity analyses conducted to assess the robustness of the synthesized results. | Sections 2.3.1, 2.3.4, 2.3.8, etc. (detailed report on the results of the sensitivity analysis) |
| Reporting biases | 21 | Present assessments of risk of bias due to missing results (arising from reporting biases) for each synthesis assessed. | Section 2.3.1 (Assessing publication bias using a funnel plot); other synthesis results were not systematically assessed |
| Certainty of evidence | 22 | Present assessments of certainty (or confidence) in the body of evidence for each outcome assessed. | Not clearly described (the certainty of the evidence was not assessed using methods such as GRADE) |
| **DISCUSSION** | | |  |
| Discussion | 23a | Provide a general interpretation of the results in the context of other evidence. | Sections 3.1, 3.2, 3.3 (Compare and interpret the results with other evidence) |
|  | 23b | Discuss any limitations of the evidence included in the review. | Section 3.4 (discusses the limitations of the evidence, such as residual confounding, interference from anticoagulant therapy, etc.) |
|  | 23c | Discuss any limitations of the review processes used. | Section 3.4 (discusses the limitations of this review process, such as insufficient subgroup stratification and not analyzing dose-response relationships) |
|  | 23d | Discuss implications of the results for practice, policy, and future research. | Sections 3.4 and 4 Summary (Discussed the implications of the results for clinical practice and directions for future research) |
| **OTHER INFORMATION** | | |  |
| Registration and protocol | 24a | Provide registration information for the review, including register name and registration number, or state that the review was not registered. | Section 1 Methods (first paragraph): This study has been prospectively registered on the international systematic review registration platform (PROSPERO) (registration number CRD420251186104). |
|  | 24b | Indicate where the review protocol can be accessed, or state that a protocol was not prepared. | Not explicitly stated (PROSPERO registration usually includes a protocol, but the method of access is not clearly described in the text) |
|  | 24c | Describe and explain any amendments to information provided at registration or in the protocol. | Not mentioned |
| Support | 25 | Describe sources of financial or non-financial support for the review, and the role of the funders or sponsors in the review. | Partially funded by grants (mentioned in the author contributions); however, the role of the funders in the review is not detailed. |
| Competing interests | 26 | Declare any competing interests of review authors. | Not explicitly stated (usually declared in a separate section at the end of the manuscript) |
| Availability of data, code and other materials | 27 | Report which of the following are publicly available and where they can be found: template data collection forms; data extracted from included studies; data used for all analyses; analytic code; any other materials used in the review. | Not clearly stated |

*From:*  Page MJ, McKenzie JE, Bossuyt PM, Boutron I, Hoffmann TC, Mulrow CD, et al. The PRISMA 2020 statement: an updated guideline for reporting systematic reviews. BMJ 2021;372:n71. doi: 10.1136/bmj.n71. This work is licensed under CC BY 4.0. To view a copy of this license, visit <https://creativecommons.org/licenses/by/4.0/>
